# Tetracycline, Macrolide and Lincosamide Resistance in *Streptococcus canis* Strains from Companion Animals and Its Genetic Determinants

**DOI:** 10.3390/antibiotics11081034

**Published:** 2022-07-31

**Authors:** Ilona Stefańska, Ewelina Kwiecień, Magdalena Kizerwetter-Świda, Dorota Chrobak-Chmiel, Magdalena Rzewuska

**Affiliations:** Department of Preclinical Sciences, Institute of Veterinary Medicine, Warsaw University of Life Sciences, Ciszewskiego 8 St., 02-786 Warsaw, Poland; magdalena_kizerwetter_swida@sggw.edu.pl (M.K.-Ś.); dorota_chrobak_chmiel@sggw.edu.pl (D.C.-C.); magdalena_rzewuska@sggw.edu.pl (M.R.)

**Keywords:** acquired-resistance genes, antimicrobial resistance, antimicrobial susceptibility testing, beta-hemolytic streptococci, companion animals, *Streptococcus canis*, zoonotic agent

## Abstract

Growing antimicrobial resistance (AMR) in companion-animal pathogens, including *Streptococcus canis* (*S. canis*), is a significant concern for pet treatment as well for public health. Despite the importance of *S. canis* in veterinary and human medicine, studies concerning the AMR of this bacterium are still scarce. A total of 65 *S. canis* strains, isolated from dogs and cats, were assessed to test for susceptibility to six clinically relevant antimicrobials via a microdilution method. The prevalence of the selected acquired-resistance genes was also investigated via PCR. High MIC_50_ and MIC_90_ values (≥128 μg/mL) were noted for tetracycline, erythromycin and clindamycin. Only a few strains were resistant to the tested beta-lactams (6.2%). Tetracycline resistance was found in 66.2% of the strains. Resistance to erythromycin and clindamycin (ML resistance) was found in 55.4% of the strains. Strains with a phenotype showing concurrent resistance to tetracycline and ML were predominant (53.8%). AMR in the tested *S. canis* strains was associated with a variety of acquired and potentially transferable genes. Tetracycline resistance was conferred by *tet*(O) (40.0%), *tet*(M) (9.2%), and *tet*(T) (1.5%), which is reported for the first time in *S. canis*. In most cases, the *tet*(M) gene was detected in relation to the conjugative transposon Tn*916*. The MLS_B_ phenotype was confirmed in the strains harboring *erm*(B) (43.1%) and *erm*(TR) (7.7%). To conclude, a high rate of *S. canis* strains occurring in dogs and cats displayed resistance to antimicrobials important for treatment; moreover, they are a potential reservoirs of various resistance determinants. Therefore, AMR in these pathogens should be continuously monitored, especially regarding the One Health concept.

## 1. Introduction

*Streptococcus canis* (*S. canis*) is a large-colony-forming, beta-hemolytic, Lancefield group G streptococci (GGS), and a member of the pyogenic group [1]. These bacteria colonize the skin and mucosal surfaces of upper respiratory tract, oropharynx, urogenital tract and perianal region in companion animals [2,3,4], mink [5] and many other mammals [6]. *S. canis* is also the most common canine streptococcal pathogen; it causes a wide spectrum of infections, including otitis externa, dermatitis, respiratory and urogenital tract infections, endocarditis and septicemia; moreover, in new-born puppies, it causes necrotizing fasciitis (NF) and streptococcal toxic shock syndrome (STSS) [7,8,9,10,11]. Similarly, in cats, *S. canis* is responsible for pyogenic infections, and the bacteria have been isolated from skin ulcerations, urogenital and upper respiratory tract infections, arthritis, sinusitis, meningitis, NF and neonatal septicemia [3,4,12]. Moreover, *S. canis* has also been described as a rare cause of subclinical mastitis in dairy cows [6].

*S. canis* is recognized as a zoonotic pathogen with increasing worldwide importance and is mainly transferred directly from companion animals to humans. The contamination of local wounds or ulcers [13] via a close contact with dogs [14] is probably a main route of the infection, and cases of bacteraemia following a dog bite have also described [15]. In humans, *S. canis* can cause mild-to-severe invasive infections, mainly including cutaneous and soft-tissue infections (e.g., ulcers) [13], urinary infections [13], osteoarticular infections [13], pneumonia [13], peritonitis [16], endocarditis [14,17], meningitis [18], bacteraemia [13,15,19] and septicemia [13,20].

The *S. canis* infections are noted to be relatively rare due to the limitations of the routine diagnostics of streptococcal infections, which is focused mainly on the hemolytic activity of an isolate and its serotyping; thus, most isolates are reported as beta-hemolytic streptococci, and hence, the frequency of *S. canis* infections seems likely to be underestimated [1,13,17,21,22]. Despite the emerging role of this pathogen, data considering the antimicrobial susceptibility of *S. canis* and genetic determinants of the observed resistance are scarce. The occurrence of antimicrobial resistance (AMR) among zoonotic bacteria is of particular concern due to the possible transmission of resistant strains from animals to humans through a variety of routes, as well as the possibility of the spread of mobile resistance determinants among different human pathogens. This is especially important because many antimicrobial agents used in an animal prophylaxis and treatment belong to the antimicrobials used in human medicine. Therefore, the consequences of the AMR of zoonotic pathogens, although quite difficult to estimate, may be far-reaching and include, for example, increased disease severity, treatment failures and the associated increased morbidity and mortality, as well as higher costs of disease treatment in both animals and humans [23]. Faced with this reality, the monitoring of AMR among important zoonotic pathogens such as *S. canis* is urgently needed, since it enables the use of most effective antimicrobial agents, thereby possibly limiting the selection of resistant strains of bacterial pathogens.

This study was conducted to investigate both the phenotypic and genotypic profiles of the AMR of clinical *S. canis* strains isolated from dogs and cats.

## 2. Results

All studied *S. canis* strains (*n* = 65; Supplementary Table S1) were beta-hemolytic and belonged to the serogroup G of streptococci. The species identification of these strains was confirmed via amplification of the specific product of the expected size (263 bp) for *S. canis* in a sodA-targeted PCR assay. 

### 2.1. Antimicrobial Susceptibility Testing

The Minimum Inhibitory Concentration (MIC), MIC_50_ and MIC_90_ values of the tested antimicrobial agents for all the studied strains are presented in Table 1. The MIC ranges for particular antibiotics are as follows: for tetracycline: 2–>128 mg/L, for penicillin G: <0.25–4 mg/L, for cephalothin: <0.25–8 mg/L, for erythromycin: <0.25–>128 mg/L, for clindamycin: <0.25–> 128 mg/L and for gentamicin: 4–128 mg/L (Table 1).

According to the used breakpoints, 21 out of 65 *S. canis* strains (32.3%; CI95%: 21.2–45.1%) (18 strains from dogs and 3 from cats) were susceptible to all the tested antimicrobial agents. A total of 8 strains (12.3%, CI95%: 5.5–22.8%) were resistant to one of the tested antimicrobials (tetracycline), whereas 36 strains (55.4%, CI95%: 42.5–67.7%) displayed resistance to more than one of the investigated antimicrobials belonging to different antimicrobial classes (1 strain to 2 antibiotics, 31 strains to 3 antibiotics, and 4 strains to 4 antimicrobial agents) (Table 2). The *S. canis* strains considered multidrug-resistant (MDR; resistance to ≥1 agent in >3 antimicrobial categories) represented 53.8% (CI95%: 41–66.3%) of all strains [24]. Eighteen strains exhibited the intermediate resistance for at least one antimicrobial agent (27.7%, CI95%: 17.3–40.2%).

**Figure 1:**
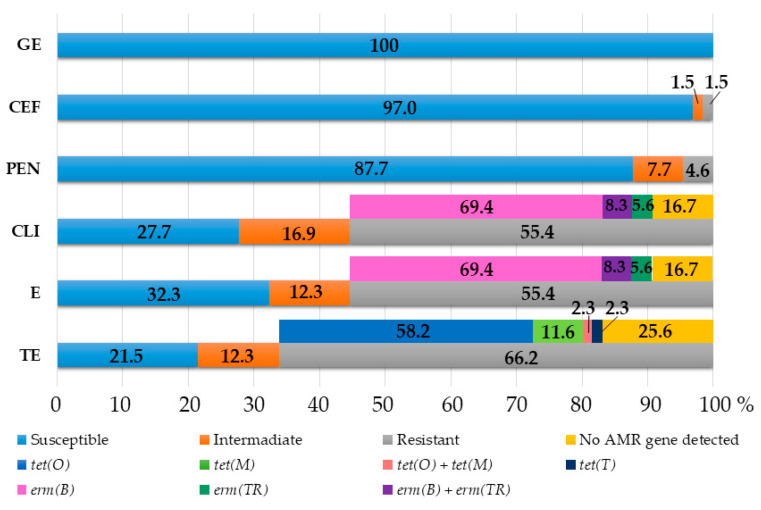
The overall rate of susceptibility and resistance to antimicrobials and AMR gene detection in 65 *S. canis* strains. GE—gentamicin, CEF—cephalothin, P—penicillin G, CLI—clindamycin, E—erythromycin, TE—tetracycline.

Five different phenotypes were observed among the tested strains (Table 2). The most common phenotype was resistant to tetracycline, erythromycin and clindamycin (31 strains). The highest frequency of resistance was recorded for tetracycline, since 43 strains were resistant (66.2%, CI95%: 53.4–77.4%), with MIC values above the breakpoint (MICs > 8) and 8 strains (12.3%, CI95%: 5.5–22.8%) being intermediate (Figure 1). ML resistance (resistance to macrolides and lincosamides) was the second most common AMR phenotype found in the studied *S. canis* strains. ML resistance to erythromycin (MIC > 4 mg/L) and clindamycin (MIC ≥ 4 mg/L) was linked in all 36 strains (55.4%, CI95%: 42.5–67.7%). Moreover, three strains exhibited intermediate resistance to clindamycin and eight strains to both erythromycin and clindamycin (Figure 1). The majority of *S. canis* strains were susceptible to all the tested beta-lactams (93.9%, CI95%: 85–98.3%), only three strains (4.6%, CI95%: 0.96–12.9%) were phenotypically resistant to penicillin G (MIC ≥ 2 mg/L), and one strain (1.5%, CI95%: 0.04–8.3%) was resistant to cephalothin (MIC ≥ 8 mg/L). Intermediate resistance to penicillin and cephalothin was noted in five strains (7.7%, CI95%: 2.6–17.1%) and one strain (1.5%, CI95%: 0.04–8.3%), respectively. No strains demonstrated a high level of resistance to gentamicin (MIC > 500mg/L) (Figure 1).

### 2.2. Detection of Tetracycline, Macrolide and Lincosamide Resistance Genetic Determinants

To identify the determinants responsible for the tetracycline and ML resistance phenotypes, the strains were screened via PCR for the presence of the selected AMR genes. Thirty-seven (56.9%, CI95%: 44–69.2%) of the strains were positive for at least one of the tested acquired AMR genes. In Table 2 the resistance phenotypes and genotypes among the 65 tested *S. canis* strains were compared. Among 43 tetracycline-resistant *S. canis* strains, the *tet*(O), *tet*(M) and *tet*(T) genes encoding ribosomal protection proteins were found in 26 strains (60.5%, CI95%: 44.4–75%), 6 strains (14%, CI95%: 5.3–27.9%) and 1 strain (1.5%), respectively. One strain carried two tetracycline-resistance genes (*tet*(O) and *tet*(M)). Five of the six *tet*(M)-positive strains carried the *xis-Tn* gene of the Tn*916* conjugative element, and all strains were negative for the *tndX* gene of the Tn*5397* transposon and the *int* gene of the Tn*5801* transposon. No strains were positive for the *tet*(W), *tet*(S), *tet*(K) or *tet*(L) genes. The *erm*(B) and *erm*(TR*)* genes were detected in 28 (77.8%, CI95%: 60.9–89.9%) and 5 (13.9%, CI95%: 4.7–29.6%) strains with the ML phenotype, respectively. Three strains carried both *erm*(B) and *erm*(TR). The *erm*(A) and *erm*(C) genes were not detected, and no strains resistant to clindamycin carried the *lnu*(B) gene. No resistance determinants were detected in the intermediate strains.

The antimicrobial-resistance phenotypes and genotypes were not consistent in 11 out of 43 tetracycline-resistant strains (25.6%, CI95%: 13.5–41.2%) and in six out of 36 ML-resistant strains (16.7%, CI95%: 6.4–32.8%), in which the corresponding AMR genes were not detected (Figure 1).

## 3. Discussion

The genus *Streptococcus* includes many commensal species, pathogens and opportunistic pathogens of humans and animals. The main streptococci of veterinary relevance tested for AMR are the bovine mastitis pathogens *Streptococcus uberis* and *Streptococcus dysgalactiae* [25]. However, the presence of antimicrobial-resistant strains in companion animals may also be important to human health. Many studies have shown that companion animals worldwide, including in Poland, can be carriers of drug-resistant bacteria, including multidrug-resistant strains such as extended-spectrum β-lactamase (ESBL) or carbapenemases-producing *Enterobacterales* and methicillin-resistant *Staphylococcus pseudintermedius* (MRSP) [26,27,28,29,30,31]. *S. canis* is one of the streptococcal pathogens most frequently isolated from various types of infection in companion animals; it is increasingly reported as a zoonotic agent, and should, therefore, be well characterized and monitored for antimicrobial resistance [3,9,22,32,33,34]. Nevertheless, data on the antimicrobial susceptibility of this bacterium are limited. To date, most of the studies have focused on the determination of the resistance phenotypes of strains [32,35,36,37,38,39], and few studies have also described the genetic resistance determinants [10,34,40,41]. Although, various methods have been used to determine antimicrobial susceptibility, the broth-dilution method is the most commonly used for the testing of *S. canis* [32,34,36,37,38,39]. However, a significant problem in the case of testing for susceptibility of streptococci isolated from animals is the lack of specific criteria for interpreting the obtained results, which make data analysis and comparison difficult and impractical. Based on the ‘One Health’ concept, the characterization of AMR in bacterial pathogens that have potential for transmission between humans and companion animals is essential for the maintenance of the health of both pets and their owners [33]. 

In our study on *S. canis* strains, the highest AMR rate was noted for tetracycline (66.2%), as well as for erythromycin and clindamycin together (55.4%). A high rate of resistance to these antimicrobial agents was also noted previously in the *S. canis* strains isolated from mink (97% for tetracycline and 53% for erythromycin, respectively) [38]. Most previous studies revealed high and dominant tetracycline resistance, ranging between 27–50%, among *S. canis* strains isolated from dogs and cats [10,34,35,36,37,40], as well as among other streptococcal species isolated from animals (38.2–100%) [25,42,43,44,45,46,47,48]. In our study, resistance to tetracycline was due to the presence of various *tet* genes, which encode a protein that protects bacterial ribosomes from the action of tetracyclines (*tet*(O), *tet*(M) and *tet*(T)). This is in line with data from previous literature, according to which the *tet*(O) and *tet*(M) genes were the most prevalent in *S. canis* [10,34,40,41] as well as in other streptococci of animal origin [25,33,40,41,42,43,46,47,49]. The *tet*(M) gene seems to be harbored by strains with the widest host range; it was previously found in numerous Gram-positive and Gram-negative species of aerobic and anaerobic bacteria, which may be due to its common association with conjugative transposons, particularly the Tn*916*–Tn*1545* family [50]. Another gene encoding ribosomal protection proteins (RPPs), but less frequently reported in *S. canis* strains, was *tet*(S) [10,25,33,40,41,49]. However, in other streptococci isolated from animals, *tet*(W) [51,52], *tet*32 [53], *tet*44 [52] and the mosaic gene *tet*(O/W/32/O) [51,52] were reported. To the best of our knowledge, this is the first study which reports the presence of the *tet*(T) gene in *S. canis*. This gene was detected using the universal primers for the detection of various *tet* genes encoding RPPs. However, it was not detected with the use of the *tet*(T)-specific primers described in other papers [54]; this may be due to some differences in the *tet*(T) sequence in *S. canis*, which may impede the detection of this gene via PCR. The amino acid (aa) sequence of Tet(T) shares 92.5% aa identity with the reference sequence of Tet(T) of *Streptococcus pyogenes* (GenBank accession no. AAF01499.1) (CARD-RGI tool, https://card.mcmaster.ca/analyze/rgi, accessed on 29 June 2022). However, according to the BLAST analysis, a 99.8% nucleotide identity to *tet*(Q) from *Helcococcus kunzii* UCN99 (KU612222.1) was found. Our analysis showed that the sequence of the Tet protein (ANZ79471.1) coded by *H. kunzii* (KU612222.1) was misidentified, and currently, the Tet protein from *H. kunzii* has a 93.1 % aa identity to Tet(T) and only a 47.0% aa identity to Tet(Q) (CARD-RGI tool). In this study, the use of newly designed primers, tetT-for and tetT-rev, enabled the detection of *tet*(T) in one *S. canis* strain, confirming the positive results obtained previously with the universal primers for RPP genes. The *tet*(T) gene was previously only found in a few bacterial species: *S. pyogenes*, *S. dysgalactiae* subsp. *equisimilis*, *Streptococcus agalactiae*, *Staphylococcus aureus*, *Staphylococcus epidermidis*, *Stenotrophomonas maltophilia*, *Lactobacillus* spp., *Clostridium difficile*, *Enterococcus faecalis* and *Pseudomonas* spp. [53,55,56,57,58,59,60].

The *tet*(K) and *tet*(L) genes encoding the energy-dependent membrane-associated proteins, which export tetracyclines out of the bacterial cell (efflux proteins) [50], were not identified in any strains tested in this study. In *S. canis*, the *tet*(L) and *tet*(K) genes were detected previously, although mostly with very low prevalence [10,33,34,40]. In *S. dysgalactie*, the presence of both the *tet*(L) [45,48] and *tet*(K) genes [43,45,47], as well as the *tet*(D) gene [48], was reported. According to the CARD database, other genes encoding the efflux pump proteins, *tet*(B), *tet*(C), *tet*(H), *tet*40 and *tet*45, were also identified in streptococci of animal origin [53].

Eleven strains with tetracycline-resistant phenotypes were negative for all the tested *tet* genes. This could be due to either potential differences in the sequences of the *tet* genes impeding their detection via PCR, or to the presence of another tetracycline-resistance determinant not investigated in this study. Various tetracycline-resistance mechanisms and related genetic determinants have been described, and the detection of each of these mechanisms requires special considerations. Currently, 63 distinct *tet* and *otr* genes, whose products have ≤80% amino acid sequence identity, have been recognized. These genes include 36 genes encoding ATP-dependent efflux proteins, 13 genes encoding RPPs, 13 genes encoding inactivating enzymes, and 1 gene conferring resistance via an unknown mechanism [61]. Moreover, eleven mosaic ribosomal protection genes resulting from the recombination between wild-type genes have been discovered [61]. According to the CARD, the prevalence of *tet* genes among the sequenced *S. canis* genomes and whole-genome shotgun (WGS) assemblies, available at NCBI, was 33.3% and 7.1% for *tet*(M), and 16.7% and 7.1% for *tet*(O) and *tet*(S), respectively [53], based on sequence data acquired from NCBI IslandViewer 4 on 7 January 2022.

Tetracycline resistance genes are often associated with mobile elements, plasmids and/or transposons and conjugative transposons facilitating horizontal gene transfer in bacteria [50,62]. Many conjugative transposons carrying different *tet* genes have been identified, and the most common are the conjugative transposon Tn*916*–Tn*1545* family, mainly associated with the *tet*(M) gene [50,62]. This conjugative element could also carry additional resistance determinants such as the erythromycin-resistance gene *erm*B, which confers resistance to macrolides, lincosamides and streptogramins B (MLS_B_ phenotype), as well as genes determining resistance to chloramphenicol and kanamycin [42,62,63]. In this study, the *tet*(M) gene was linked to the *erm*(B) gene in two strains (65/22 and 53/21), and with the *erm*(TR) gene in one strain (3/16). Importantly, it has been shown that Tn*916*-like transposons could be transferred to many different species with a relatively high transfer frequency [62,63]. These findings highlight the role of *S. canis* in the spread of antimicrobial-resistance determinants within and across bacterial species. A Tn*916*-related element has also been detected in other tetracycline-resistant streptococci, *S. agalactiae*, *S. uberis* and *S. dysgalactiae*, with strains carrying the Tn*916*-related transposon and the *tet*(S) gene [42]. *tet*(M) has also been found in other conjugative transposons: Tn*5397* and Tn*5801* [62,64]; however, these mobile elements were not found in any of the six *tet*(M)-positive strains in this study. This suggests that the tetracycline-resistant *S. canis* 18/16 strain without the transposons carried *tet*(M), probably on a plasmid. In contrast to the *tet*(M) gene, *tet*(O) and *tet*(T) are not associated with conjugative transposons but can be mobile when carried by conjugative plasmids [53,62].

A high rate of resistance against macrolides and lincosamides in *S. canis* strains was reported, which is consistent with the study by Moyaert et al. (69.6% and 23.26%, respectively) [35]. In numerous studies, the prevalence of resistant strains was lower and noted to be approximately between 2.4% and 23%, and 2.4% and 16%, respectively [10,34,35,36,40,65]. Similar findings were reported for other streptococcal species important in veterinary medicine. Some authors showed high macrolide resistance in *S. dysgalactiae*, noted to be 60.0% [46], 57% [38], 43.8% [45] or 36.7% [48], and in *S. uberis*, reported to be 74.3% [42]. However, in some studies, almost 90% of the strains of *S. dysgalactiae* and *S. agalactiae* were susceptible to macrolides [42,43,47]. Similarly, for lincosamides, the prevalence of resistance phenotypes was between 5.5% and 56% [44,45,47,48]. Both the constitutive macrolide/lincosamide/streptogramin B (cMLS_B_) and the inducible macrolide/lincosamide/streptogramin B (iMLS_B_) resistance phenotypes were previously found in streptococci [10,40,41,43]. In this study, strains presenting the cMLS_B_ phenotype were conferred by different *erm* genes, *erm*(B) and *erm*(TR), which are variants of *erm*(A). To the best of our knowledge, it seems that *erm*(TR) has not been previously described in *S. canis*. *erm* genes encoding rRNA methylases, which modify the ribosomal target site, are the most common mechanism of MLS_B_ resistance in streptococci, often carrying by plasmids and conjugative transposons [62,66,67,68]. The resistance determined by the *erm* genes (*erm*(B) and *erm*(A) and *erm*A(TR)) was also the most frequently detected in previous studies on *S. canis* [10,34,40,41] and other streptococci isolated from animals and humans [42,43,47,48,56,68,69]. According to the CARD base, the prevalence of *erm*(B) among the 14 WGS assemblies available at NCBI for *S. canis* was 14.29%. In this study, six *S. canis* strains with the cMLS_B_ phenotype were negative for the tested *erm* genes. This indicates the presence of other genes conferring resistance to macrolides and/or lincosamides, not detected in this study; this is not surprising considering the significant genetic diversity of resistance determinants. Some authors noted a significantly lower resistance to erythromycin than to lincomycin, suggesting the presence of a non-*erm*-mediated mechanism of resistance [25]. Currently, 124 distinct genes conferring MLS_B_ resistance have been recognized. These genes include 47 *erm* genes encoding rRNA methylases, 8 genes encoding efflux pumps, 32 genes encoding ABC-F proteins that confer resistance via ribosomal protection (for 6 genes, only a sequence and aa support this mechanism), and 37 genes encoding inactivating enzymes, including 4 esterases, 2 lyases, 16 transferases and 15 phosphorylases [66]. Thus far, other mechanisms of resistance to macrolides and/or lincosamides have also been found in streptococcal species of veterinary relevance, represented by efflux mediated by the Mef, and rarely MreA, efflux pumps belonging to the MFS family (mainly MefA) [25,42,43,45,48,52,69,70,71], and by Msr (mainly MsrD), LsaE and LsaC ABC transporters [51,52,69,71]. The inactivation of antibiotics due to Lnu transferases encoded by the *lnu*(B) (formerly *lin*(B)), *lnu*(A), *lnu*(C) and *lnu*(D) genes, and Mph phosphorylases encoded by the *mph*(B) and *mph*(C) genes, were also reported [25,42,51,52,53,71]. 

In this study, all the tested *S. canis* strains exhibited high sensitivity to the beta-lactams, which is in accordance with the majority of the data from the previous literature [10,33,34,35,36,40]. A low level of ampicillin resistance was found in *S. canis* strains in the study by Awji et al. (2012) [37]. Generally, streptococci isolated from animals are highly susceptible to beta-lactams [43,44,46,51,56,72,73], and documented resistance to beta-lactams was noted mainly in bovine streptococci [25]. Samir et al. (2020) reported the emergence of penicillin macrolide-resistant *S. pyogenes* among pet animals [74]. Based on various published studies, Bonofiglio et al. (2018) determined the median MIC_90_ values of beta-lactams for group A, C and G streptococci as 0.016 µg/mL (range 0.0025–0.032 µg/mL) [75]. This phenomenon is surprising as beta-lactams are often prescribed as the drug of choice for the treatment of many streptococcal infections [51,52,73]. The most common and most important mechanism of antimicrobial resistance to beta-lactams is the expression of antibiotic-inactivating enzymes, beta-lactamases, which are one of the most numerous enzyme families. Over 1300 beta-lactamases have been recognized, including ESBLs, cephalosporinases (AmpCs) and carbapenemases, and these have become a major concern [76]. In addition to the production of beta-lactamases, resistance to these antimicrobial agents can also be due to the modification of penicillin-binding proteins (PBPs) [67]. In streptococci, rarely reported in the literature, beta-lactam resistance was associated with both mechanisms, the presence of modified PBPs [25,51,73], and the production of the BlaZ beta-lactamase; however, the presence of *blaZ* did not always correspond with phenotypic resistance to beta-lactams [72,77].

All the *S. canis* strains were susceptible to gentamicin. For gentamicin, streptomycin and kanamycin, MIC ≤ 250 μg/mL was considered intrinsic low-level resistance, whereas MIC > 500 μg/mL indicated the presence of acquired resistance to aminoglycosides [78]. Importantly, a low level of resistance to aminoglycosides do not prevent the bactericidal synergistic effect between aminoglycosides and penicillin [78].

The main limitation of the current study is the small number of *S. canis* strains tested; moreover, the samples represent their geographically limited distribution. Another important issue is the lack of veterinary-specific interpretation criteria for *S. canis* to determine whether a strain is susceptible or resistant to a given antimicrobial agent. The used breakpoint values have a crucial impact on the results of susceptibility testing. Regarding these limitations, we have shown that the AMR of *S. canis* to tetracycline and MLS is common and should be taken into consideration in small-animal veterinary practice. This study highlights the relevance of further investigation to provide susceptibility results for *S. canis* strains isolated from animals, as well as to assess or improve microbiological breakpoints.

## 4. Materials and Methods

### 4.1. Bacterial Strains

A total of 65 *S. canis* strains from companion animals (dogs, *n* = 55 and cats, *n* = 10) were tested. All strains were recovered from clinical specimens, taken from animals with different types of infections, at the Microbiological Diagnostic Laboratory, Institute of Veterinary Medicine, Warsaw University of Life Sciences, Poland (Supplementary Table S1). Sampling sites were as follows: urogenital tract samples (*n* = 29), skin and soft-tissue infection (*n* = 6), internal organs (*n* = 5), respiratory tract (*n* =5), ear (*n* = 5), conjunctival swabs (*n* = 4), oral cavity/periodontium (*n* = 6) and others (*n* = 5) (Supplementary Table S1).

The animals belonged to different owners and there was no evident epidemiologic relationship. Bacteria were cultivated on Columbia agar supplemented with 5% sheep blood (CA) (Graso Biotech, Starogard Gdański, Poland) at 37 °C for 24h under aerobic conditions. All tested strains were primarily identified as *Streptococcus* spp. by observing phenotypic features such as Gram staining, growth and cell morphology; this included the type of hemolysis on CA and basic biochemical tests (oxidase and catalase activities). The *S. canis* strains were identified to the species level using the MICROGEN^®^Strep (M47) latex agglutination test (Microgen Bioproducts Ltd., UK) and PCR with canis-sod-I and canis-sod-II primers, previously described by Hassan et al. (2005) [6]. All strains were stored at −20 °C in a tryptic soy broth (Graso Biotech, Starogard Gdański, Poland) containing 20% glycerol (Sigma-Aldrich, Steinheim, Germany).

### 4.2. Antimicrobial Susceptibility Testing

In this study, *S. canis* strains were tested against antimicrobial agents belonging to five different functional classes. The antimicrobials were: penicillin G and cephalothin (β-lactams), gentamicin (aminoglycosides), tetracycline (tetracyclines), erythromycin (macrolides) and clindamycin (lincosamides). Clindamycin was manufactured by the European Pharmacopoeia Reference Standards, while other antibiotics were manufactured by Sigma-Aldrich (Sigma-Aldrich, Steinheim, Germany). The strains were tested using the broth microdilution method, according to the CLSI guidelines [79]. All antimicrobials were diluted in Müeller–Hinton broth (Graso Biotech, Starogard Gdański, Poland) supplemented with 5% (*v*/*v*) horse serum (Graso Biotech, Starogard Gdański, Poland) (MHB) to obtain a final concentration in the range of 0.125 µg/mL to 128 µg/mL (two-fold serial dilutions). A bacterial suspension, equivalent to the 0.5 McFarland standard, was prepared in MHB using the colonies obtained from an overnight culture on CA (aerobic incubation, 37 °C, 18–22 h). The lowest concentrations of each antimicrobial agent that inhibited the visible growth of bacteria (MIC, Minimum Inhibitory Concentration), were determined after 24 and 48 h of incubation at 37 °C under aerobic conditions. The antimicrobial concentrations required to inhibit the growth of 50% (MIC_50_) and 90% (MIC_90_) of the strains were also determined. The MIC breakpoints that were used in this study to classify strains as susceptible or resistant are listed in Supplementary Table S2. The MIC breakpoints for penicillin G, cephalothin and clindamycin were based on the interpretative criteria recommended for beta-hemolytic *Streptococcus* spp. of canine origin or *Streptococcus* spp. of equine origin, as defined by the current CLSI guidelines VET08 [79]. However, there are no breakpoints available in these guidelines for tetracycline, gentamicin and erythromycin specific to the *Streptococcus* spp. beta-hemolytic group [79]. Thus, the susceptibility to those antimicrobial agents was based on the interpretative criteria recommended for *Streptococcus* spp. in accordance with the Antibiogram Committee of the French Microbiology Society (CA-SFM) guidelines Vet 2021 (https://www.sfm-microbiologie.org/wp-content/uploads/2021/12/CASFM_VET2021.pdf, accessed on 29 June 2022) [78]. The accuracy of antimicrobial susceptibility testing was controlled using two reference strains, *Escherichia coli* ATCC 25922 and *S. aureus* ATCC 25923. 

### 4.3. Detection of Selected Resistance Genetic Determinants

All *S. canis* strains phenotypically resistant to tetracycline were examined via PCR assay for the presence of genes encoding ribosomal-protection proteins (first with the universal primer set, and subsequently for positive strains, with specific primers for *tet*(M), *tet*(O) and *tet*(T) genes), as well as the *tet*(K) and *tet*(L) genes encoding a tetracycline efflux pump. The strains harboring the *tet*(M) gene were screened for the presence of the transposons Tn*916*, Tn*5801* and Tn*5397* linked with this gene, and also for the region of excisionase (the *xis* gene), integrase (the *int* gene) and resolvase (the *tndX* gene) associated with these elements, respectively. All strains demonstrating the MLS_B_ phenotype (erythromycin and clindamycin-resistant strains) were screened to detect the *erm*(A), *erm*(TR), *erm*(B) and *erm*(C) genes. Additionally, the *lnu*(B) gene (a determinant of the lincosamide resistance) was tested. PCR mixtures contained 1 µL of each primer (10 pmol/µL), 12.5 µL of DreamTaq PCR Master Mix (2×) (Thermo Fisher Scientific, Waltham, MA, USA), 40 ng of DNA and water up to 25 µL. All primers were synthesized by Eurofins Genomics Germany GmbH (Ebersberg, Germany) and are listed in Supplementary Table S3 [6,42,54,55,80,81,82,83,84,85,86,87]. To extract a DNA template, several colonies were picked from a bacterial culture on CA and were suspended in 500 µL of water-free of DNase. The suspension was boiled in a water bath for 10 min and kept on ice for a few minutes; after that, cellular debris was removed via centrifugation at 12,000 ×*g* for 10 min. The supernatant was use as a DNA template. The DNA concentration was estimated spectrophotometrically (NanoDrop, 1000 Spectrophotometer, Thermo Fisher Scientific, Waltham, MA, USA) and the DNA samples were stored at −20 °C until further analysis. 

The PCR products were separated on 1.0% (*m*/*v*) agarose gel containing MidoriGreen (Nippon Genetics, Düren, Germany). The amplicons were visualized under UV light (Gel Doc^TM^ EZ Imager, Image Lab ver. 5. 2. 1. software, BioRad, Hercules, CA, USA). The GeneRuler^TM^ 100bp Plus DNA Ladder (Thermo Fisher Scientific, Waltham, MA, USA) was used as a standard size marker. In cases of a positive result with the universal DI_F and DII_R primers, but negative with the primer set specific for particular tetracycline-resistance genes, the amplicon was sequenced to determine the types of *tet* genes. The nucleotide sequence of the *tet*(T) gene of strain 52/21, firstly described in *S. canis*, was analyzed using Chromas 2.6.5 software (http://www.technelysium.com.au/chromas.html, accessed on 29 June 2022). The sequence was identified using bioinformatics tools including BLAST (Basic Local Alignment Search Tool, http://blast.ncbi.nlm.nih.gov/Blast.cgi, accessed on 29 June 2022) and the Comprehensive Antibiotic Resistance Database–Resistance Gene Identifier software (CARD-RGI, https://card.mcmaster.ca/analyze/rgi, accessed on 29 June 2022). The nucleotide sequence for *tet*(T) was submitted to the GenBank (accession no. OM973245).

### 4.4. Development of New Primers for Tet(T) Detection

A new primer set was developed to detect the *tet*(T) gene in *S. canis*. The primers tetT-for and tetT-rev were designed using the PCR Primer Design Tool (https://eurofinsgenomics.eu/en/ecom/tools/pcr-primer-design/, accessed on 29 June 2022) and were checked for the formation of self-dimers and cross-dimers using an Oligo Analysis Tool (https://www.eurofinsgenomics.eu/en/ecom/tools/oligo-analysis, accessed on 29 June 2022).

### 4.5. Statistical Analysis

Confidence intervals were calculated using the online Sample Size Calculator tool [88].

## 5. Conclusions

In conclusion, the presented data show that *S. canis* strains isolated from dogs and cats are resistant to antimicrobial agents commonly used in veterinary and human medicine practice. Forty-four strains (67.7%) were resistant to at least one antimicrobial, and thirty-seven strains (56.9%) harbor a variety of acquired and potentially transferable genes that conferred resistance to tetracyclines (*tet* genes) and MLS_b_ antibiotics (*erm* genes). These genes are often related to various mobile genetic elements, such as conjugative transposon Tn*916* linked to *tet*(M), found in five *S. canis* strains. In our study, resistance phenotypes and genotypes were not consistent in some cases. Therefore, further investigations, conducted on larger number of strains, are needed to estimate new breakpoints and to discover other determinants of AMR in this species. However, the presented results allow a better insight into the resistance of *S. canis*, one of the most important zoonotic streptococcal pathogens occurring in companion animals.

## Figures and Tables

**Table 1:** Distribution of Minimum Inhibitory Concentration (MIC) of six antimicrobial agents, MIC_50_ and MIC_90_ values for the studied *S. canis* strains from cats (*n* = 10) and dogs (*n* = 55).

Animal	Antibiotic *	Number of Strains with MIC (μg/mL):										MIC_50_	MIC_90_
		≤0.25	0.5	1	2	4	8	16	32	64	≥128		
Cat	PEN	9		1								≤0.25	≤0.25
	CEF	5	2	2		1						≤0.25	1
	GE					1	4	5				8	16
	TE				1		2				7	≥128	≥128
	E	2	1								7	≥128	≥128
	CLI		3								7	≥128	≥128
Dog	PEN	45	3	4	2	1						≤0.25	1
	CEF	28	18	6	2		1					≤0.25	1
	GE					1	12	33	7	1	1	16	32
	TE				1	12	6	2	1	4	29	≥128	≥128
	E	15	4	4	1	2		1			28	≥128	≥128
	CLI	9	9	5	3		1				28	≥128	≥128

^*^ Antimicrobial agents used in this study: penicillin G (PEN), cephalothin (CEF), gentamicin (GE), tetracycline (TE), erythromycin (E) and clindamycin (CLI). Light-grey shading indicates strains displaying an intermediate phenotype based on the breakpoints defined in Supplementary Table S2; Dark-grey shading indicates strains displaying a resistant phenotype based on the breakpoints defined in Supplementary Table S2.

**Table 2:** The consistency between the resistance phenotype and genotype among studied *S. canis* strains (*n* = 44).

Strain	Resistance Phenotype ^1^	Resistance Genes Detected
12/16	TE	n.d. ^2^
22/18		n.d.
27/18		*tet*(O) ^3^
35/20		n.d.
44/21		*tet*(O) ^3^
1/16		*tet*(M) linked with Tn*916*-like transposon
3/16		*tet*(M) linked with Tn*916*-like transposon
52/21		*tet*(T)
31/20	E-CLI	*erm*(B)
14/16	TE-E-CLI	*tet*(O), *erm*(B), *erm*(TR)
15/16		*tet*(O), *erm(*B), *erm*(TR)
18/16		*tet*(M) ^3^, *erm*(B)
23/18		*erm*(B)
24/18		*tet*(O), *erm*(B)
32/20		*tet*(O) ^3^, *erm*(B)
48/21		*tet*(O) ^3^
51/21		*tet*(O), *erm*(B)
58/21		*erm*(B)
60/21		*tet(O)*, *erm*(B)
2/16	TE-E-CLI	*tet*(O), *erm*(B)
4/16		*tet*(O), *erm*(B)
5/16		*tet*(O), *erm*(B)
6/16		*tet*(O), *erm*(TR)
7/16		*tet*(O), *erm*(B), *erm*(TR)
10/16		*tet*(O), *erm*(B)
17/16		*tet*(O), *erm*(B)
19/16		n.d.
20/17		*tet*(O), *erm*(B)
25/18		*tet*(O) ^3^, *erm*(B)
39/21		*tet*(O), *erm*(B)
47/21		*tet*(O), *erm*(B)
49/21		n.d.
50/21		*tet*(M*)* linked with Tn*916*-like transposon
55/21		n.d.
56/21		*erm*(TR)
57/21		*erm*(B)
61/21		*tet*(O), *erm*(B)
62/21		*tet*(O) ^3^, *erm*(B)
63/21		*tet*(O), *erm*(B)
65/22		*tet*(M) linked with Tn*916*-like transposon, *tet*(O), *erm*(B)
59/21	TE-E-CLI-P	*tet*(O), *erm*(B)
64/21		n.d.
41/21		*tet*(O), *erm*(B)
53/21	TE-E-CLI-CEF	*tet*(M) linked with Tn*916*-like transposon, *erm*(B)

^1^ TE—tetracycline, E—erythromycin, CLI—clindamycin, P—penicillin G, CEF—cephalothin; ^2^ n.d.—tested resistance genes were not detected; ^3^ PCR assay for the presence of genes encoding ribosomal protection proteins with universal primer set (DI_F and DII_R) were negative.

## Data Availability

The data presented in this study are available in the article or supplementary materials.

## Supplementary Materials

The following supporting information can be downloaded at: https://www.mdpi.com/article/10.3390/antibiotics11081034/s1, Table S1: Characteristics of the *S. canis* strains included in this study; Table S2: MIC interpretive criteria for the tested antimicrobial agents; Table S3: Primers used in this study.
