# Supplementary material for: Tetracycline, Macrolide and Lincosamide Resistance in Streptococcus canis Strains from Companion Animals and Its Genetic Determinants
Source: Antibiotics (Basel). 2022 Jul 31;11(8):1034. doi: 10.3390/antibiotics11081034 (PMC9405182; doi:10.3390/antibiotics11081034)
Supplement: Supplementary file 1 [file antibiotics-11-01034-s001.zip › Supplementary Table S1.pdf]

Supplementary Table S1. Characteristics of the *S. canis* strains included in this study.

No.	Strains*	Host	Sources
1.	5/16	cat	urine
2.	6/16	cat	internal organs
3.	7/16	cat	urine
4.	13/16	cat	urine
5.	21/17	cat	urine
6.	32/20	cat	prepuse
7.	38/21	cat	feces
8.	50/21	cat	conjunctiva
9.	51/21	cat	conjunctiva
10.	57/21	cat	lung, spleen, liver
11.	1/16	dog	ear
12.	2/16	dog	uterine horn
13.	3/16	dog	urine
14.	4/16	dog	urine
15.	8/16	dog	urine
16.	9/16	dog	ear
17.	10/16	dog	urine
18.	11/16	dog	bile
19.	12/16	dog	abscess
20.	14/16	dog	vagina
21.	15/16	dog	larynx
22.	16/16	dog	perineum
23.	17/16	dog	chin
24.	18/16	dog	perineum
25.	19/16	dog	uterus
26.	20/17	dog	wound
27.	22/18	dog	periodontium
28.	23/18	dog	skin fistula
29.	24/18	dog	vagina
30.	25/18	dog	eye
31.	26/18	dog	lung
32.	27/18	dog	feces
33.	28/19	dog	ear
34.	29/19	dog	urine
35.	30/19	dog	ear
36.	31/20	dog	nose
37.	33/20	dog	oral cavity
38.	34/20	dog	periodontium
39.	35/20	dog	periodontium
40.	36/21	dog	feces

41.	37/21	dog	urine
42.	39/21	dog	spleen, lung
43.	40/21	dog	urine
44.	41/21	dog	nose
45.	42/21	dog	skin
46.	43/21	dog	urine
47.	44/21	dog	periodontium
48.	45/21	dog	skin
49.	46/21	dog	conjunctiva
50.	47/21	dog	periodontium
51.	48/21	dog	mitral valve
52.	49/21	dog	urine
53.	52/21	dog	vagina
54.	53/21	dog	urine
55.	54/21	dog	urine
56.	55/21	dog	prepuse
57.	56/21	dog	throat
58.	58/21	dog	ear
59.	59/21	dog	chin
60.	60/21	dog	urine
61.	61/21	dog	semen
62.	62/21	dog	semen
63.	63/21	dog	semen
64.	64/21	dog	trachea
65.	65/22	dog	urine

* The strain name includes the year of isolation
