# Supplementary material for: Tetracycline, Macrolide and Lincosamide Resistance in Streptococcus canis Strains from Companion Animals and Its Genetic Determinants
Source: Antibiotics (Basel). 2022 Jul 31;11(8):1034. doi: 10.3390/antibiotics11081034 (PMC9405182; doi:10.3390/antibiotics11081034)
Supplement: Supplementary file 1 [file antibiotics-11-01034-s001.zip › Supplementary Table S2.pdf]

Supplementary Table S2. MIC interpretive criteria for the tested antimicrobial agents.

Antimicrobial agent	Antimicrobial breakpoints (µg/mL)			Breakpoints from
	S	I	R	
Cephalothin	≤ 2	4	≥ 8	CLSI VET08, beta-haemolytic *Streptococcus* spp. from dogs
Penicillin G	≤ 0.5	1	≥2	CLSI VET08, *Streptococcus* spp. from horses
Gentmicin	≤ 250	500	> 500	CA-SFM Vet2021, *Streptococcus* spp.
Erythromycin	≤ 1	2-4	> 4	CA-SFM Vet2021, *Streptococcus* spp.
Clindamycin	≤ 0.5	1-2	≥ 4	CLSI VET08, beta-haemolytic *Streptococcus* spp. from dogs
Tetracycline	≤ 4	8	> 8	CA-SFM Vet2021, *Streptococcus* spp.

Abbreviation: S: susceptible, I: intermediate, R: resistant
