# Supplementary material for: Tetracycline, Macrolide and Lincosamide Resistance in Streptococcus canis Strains from Companion Animals and Its Genetic Determinants
Source: Antibiotics (Basel). 2022 Jul 31;11(8):1034. doi: 10.3390/antibiotics11081034 (PMC9405182; doi:10.3390/antibiotics11081034)
Supplement: Supplementary file 1 [file antibiotics-11-01034-s001.zip › Supplementary Table S3.pdf]

Supplementary Table S3. Primers used in this study.

Primer designation	Primer sequence (5′-3′)	Target gene	Annealing temperature (°C)	Amplicon size (bp)	Reference[1]
canis-sod-I<br>canis-sod-II	AGAATTATTGGCAGATGTCACTA<br>TTTCAAGTTGTCCTTCCTTATTG	*sodAint*	60	263	6
DI_F<br>DII_R	GAYACICCIGGICAYRTIGAYTT<br>GCCCARWAIGGRTTIGGIGGIACYTC	genes encoding ribosomal protection proteins	53	1100	55
TKI_F<br>TL32_R	CCTGTTCCCTCTGATAAA<br>CAAACTGGGTGAACACAG	*tet*(K)/*tet*(L)	50	1050	80
tetK_F<br>tetK_R	TATTTGGCTTTGTATTCTTTCAT<br>GCTATACCTGTTCCCTCTGATAA	*tet*(K)	50	1159	81
tetL_F<br>tetL_R	ATAAATTGTTTCGGGTCGGAAT<br>AACCAGCCAACTAATGACAATGAT	*tet*(L)	50	1077	81
tetM_F<br>tetM_R	TTAAATAGTGTTCTTGGAG<br>CTAAGATATGGCTCTAACAA	*tet*(M)	54	656	82
tetO_F<br>tetO_R	GGCGTTTTGTTTATGTGCG<br>ATGGACAACCCGACAGAAGC	*tet*(O)	50	559	83
tetT_FW<br>tetT_RV	AAGGTTTATTATATAAAAGTG<br>AGGTGTATCTATGATATTTAC	*tet*(T)	46	169	54
tetT_fwd<br>tetT_rev	GGCGATGTCATTRCAGAAAAAG<br>AGTGCTTCTATCAGTTTGCTTC	*tet*(T)	56	521	OM973245 [2]
tetS fwd<br>tetS rev	TCCGATAGTGATCCCCTTCT<br>GGAAATCTGCTGGCGTACTG	*tet*(S)	57	445	42
tetW_F<br>tetW_R	GAGAGCCTGCTATATGCCAGC<br>GGGCGTATCCACAATGTTAAC	*tet*(W)	64	168	54
Tn916-1<br>Tn916-2	GCCATGACCTATCTTATA<br>CTAGATTGCGTCCAA	Tn*916*-like (*xis-Tn*)	39	476	84
Tn5397-tndX-1<br>Tn5397-tndX-1	ATGATGGGTTGGACAAAGA<br>CTTTGCTCGATAGGCTCTA	Tn*5397*-like (*tndX*)	46	610	84
intcw459-1<br>intcw459-2	CCGATATTGAGCCTATTGATGTG<br>GTCCATACGTTCCTAAAGTCGTC	Tn*5801*-like (*int*)	58	722	85
ermA_F<br>ermA_R	TCTAAAAAGCATGTAAAAGAA<br>CTTCGATAGTTTATTAATATTAGT	*erm*(A)	52	645	86
ermB_F<br>ermB_R	GAAAAGGTACTCAACCAAATA<br>AGTAACGGTACTTAAATTGTTTAC	*erm*(B)	52	639	86
ermC_F<br>ermC_R	TACAAACATAATATAGATAAA<br>GCTAATATTGTTTAAATCGTCAAT	*erm*(C)	52	642	86
erm(TR)1<br>erm(TR)2	CTTGTGGAAATGAGTCAACGG<br>TTGTTCATTGGATAATTTATC	*erm*(TR)	48	551	87

[1]Reference or GenBank accession number of sequence taken into account for the primer design. [2]Primer set designed in this study.
